# Monocular Structured-Light Sensing for 3D Metallic Hole Measurement

**DOI:** 10.3390/s26154900

**Published:** 2026-08-03

**Authors:** Zixuan Lv, Lijie Chen, Yu Tian, Xinyue Zhang, Jinliang Shao, Yinghao Liu, Xiaoyong Lv, Jiangxiong Zhu, Zhikun Zhan, Yuliang Zhao

**Affiliations:** 1 School of Computer and Communication Engineering, Northeastern University at Qinhuangdao, Qinhuangdao 066004, China; 202312060@stu.neu.edu.cn (Z.L.); chenlijie@mails.neu.edu.cn (L.C.); 2 School of Information Science and Engineering, Northeastern University, Shenyang 110819, China; 2372313@stu.neu.edu.cn (Y.T.); zxy031544@163.com (X.Z.); 2372299@stu.neu.edu.cn (J.S.); 2410330@stu.neu.edu.cn (Y.L.); xiaoyonglv@neuq.edu.cn (X.L.); 3 School of Agriculture and Biology, Shanghai Jiao Tong University, Shanghai 200240, China; zjx261023@163.com; 4 Hebei Key Laboratory of Marine Perception Network and Data Processing, Northeastern University at Qinhuangdao, Qinhuangdao 066004, China

**Keywords:** monocular structured light, metallic circular hole, HDR fusion, sub-pixel stripe extraction, robust circle fitting, non-contact dimensional inspection

## Abstract

Non-contact metric inspection of metallic circular holes is essential for assembly quality control, yet remains difficult on reflective machined surfaces, where depth scale, stripe radiometry, and contour geometry degrade simultaneously. Pure monocular two-dimensional vision localizes hole boundaries efficiently but cannot resolve metric depth; multi-camera three-dimensional systems remove this ambiguity but with a heavier hardware and calibration cost; and conventional structured-light pipelines often improve stripe extraction or circle fitting in isolation, leaving the overall measurement chain fragile when reflection and edge defects co-occur. This paper proposes a monocular structured-light framework that treats sensing geometry, radiometric stripe reliability, and outlier-robust hole estimation as one coupled measurement chain. A single calibrated industrial camera is combined with an obliquely projected line laser to fuse top-view contour observation with light-plane-constrained depth recovery. Multi-exposure high-dynamic-range (HDR) fusion, adaptive Gaussian regularization, and distance-weighted gray-centroid refinement stabilize sub-pixel stripe centerlines under local saturation and uneven illumination, while geometry-aware contour screening and probabilistic multi-stage RANSAC fitting suppress burr-induced outliers during circle-parameter estimation. Experiments were conducted on a steel bolt-hole workpiece and a 6061 aluminum-alloy plate containing five holes with nominal diameters spanning approximately 30–78 mm. The original steel workpiece was evaluated for its diameter and two datum-related center-position quantities, while the five-hole plate was evaluated through three repeated optical diameter measurements and independent CMM references. For the five aluminum-alloy holes, the mean optical–CMM diameter differences ranged from 0.006 to 0.218 mm, with an average absolute difference of 0.096 mm. The results establish feasibility over the tested workpieces and calibrated measurement volume rather than generalization to arbitrary hole geometries, materials, or surface conditions.

## 1. Introduction

Metallic circular-hole inspection is a fundamental task in industrial quality control, with wide application in aerospace assembly, automotive body-in-white manufacturing, rail-vehicle structural fabrication, and heavy-machinery production. The objective is to recover metric geometric quantities—especially hole diameter and position relative to structural references—from non-contact optical observations. Because circular holes govern positioning, fastening, and load transfer in structural components, their dimensional consistency directly affects assembly alignment, fastening integrity, and long-term service reliability [1]. In production environments, inspection systems are expected to provide high accuracy, stable repeatability, and deployable hardware. Contact instruments, such as calipers, plug gauges, and coordinate-measuring machines, remain reliable reference tools in controlled environments. Their use in production inspection can nevertheless be limited by probe accessibility, fixturing and alignment requirements, measurement takt time, operator intervention, and the risk of contacting sensitive surfaces. Reflective, coated, or partially contaminated surfaces primarily challenge optical sensing through saturation, non-uniform illumination, and reduced stripe contrast [2,3,4]. Non-contact vision therefore offers a scalable route to in-line dimensional quality control, provided that the sensing system can recover metric three-dimensional geometry rather than image-domain appearance alone.

Existing non-contact methods have complementary strengths but still leave a critical gap for metallic circular-hole inspection. Monocular two-dimensional vision can localize hole boundaries efficiently and is easy to deploy, yet it lacks the depth sensitivity required for metric three-dimensional measurement. Multi-camera and binocular three-dimensional systems remove depth ambiguity and have reported high hole-measurement accuracy [5,6,7,8], but they increase hardware complexity, calibration burden, and sensitivity to edge artifacts. Line-structured-light sensing provides an attractive compromise by introducing an active depth cue with relatively compact hardware [9,10,11]; recent studies have improved stripe centerline localization under varying surface conditions [12,13,14,15]. Recent learning-based vision studies have also explored noise-aware feature embedding for small-object detection [16] and robust multimodal representation under cross-domain disturbance [17]; however, such data-driven pipelines generally lack explicit metric geometric constraints and calibrated structured-light models, making them difficult to deploy directly for dimensional metrology on reflective metallic workpieces. Nevertheless, most existing pipelines still optimize stripe extraction or circle fitting in isolation rather than treating sensing geometry, radiometric stripe reliability, and hole-parameter estimation as one coupled measurement chain. The core problem addressed in this work is therefore how to maintain metric three-dimensional inspection accuracy for metallic circular holes on reflective machined surfaces when the full measurement pipeline—rather than any single algorithmic stage—becomes the limiting factor.

Recent structured-light studies provide more direct reference points for circular or internal dimensional inspection. Ye et al. [18] developed a circle-structured-light inner-surface inspection system and reported a diameter absolute error below 3 μm and a defect resolution better than 0.02 mm. Dong et al. [19] compensated light-plane and stage-motion systematic errors in a circle-structured-light system and reported a mean relative measurement error of 0.015%. Zhao et al. [20] introduced a multi-diameter concentric calibrator for circular structured light and reported a relative error below 0.012%. For circular-hole measurement based on other sensing arrangements, Xia et al. [6] reported an error of approximately 0.05 mm using binocular vision, while Huang et al. [7] achieved standard deviations of 0.017 mm for chamfered-hole radius and 0.021 mm for hole spacing. For reflective metallic surfaces measured by line-structured light, Song et al. [14] reported an average standard-block height error of 0.025 mm and a repeatability value of 0.026 mm. These results were obtained for different measurands, specimens, sensing geometries, working distances, calibration procedures, and uncertainty definitions and therefore do not constitute a directly controlled ranking. In particular, the cited circle-structured-light systems mainly target inner-surface scanning, whereas the present monocular line-structured-light system integrates reflective-stripe observation, external hole-contour fitting, and datum-related dimensional extraction using one camera and one line laser.

This problem gives rise to two tightly coupled challenges. Challenge 1: Metric depth-scale recovery and radiometrically stable stripe observation under compact sensing. A practical industrial system should avoid the hardware and calibration overhead of multi-camera configurations, yet a single monocular view cannot resolve metric depth by itself. Line-structured-light triangulation can compensate for this limitation, but on machined metallic surfaces the projected stripe often suffers from local saturation, uneven width, and intensity asymmetry; under such conditions, unstable sub-pixel centerline extraction directly propagates into depth recovery errors. Challenge 2: Robust circular-hole parameter estimation from imperfect industrial contours. Even when a hole boundary is visible in the image domain, burrs, chamfers, partial edge loss, and highlight-induced outliers can corrupt circle fitting if the pipeline relies on deterministic least-squares or direct algebraic estimation [21,22,23,24,25]. Because stripe localization errors degrade depth recovery and contour fitting errors further distort diameter and position estimation, these two challenges must be solved jointly rather than independently.

To address the above challenges, this paper proposes a monocular structured-light measurement framework for metallic circular-hole inspection. The framework consists of three coordinated modules. For challenge 1, a monocular industrial camera and an obliquely projected line laser fuse top-view contour observation with light-plane-constrained depth recovery; multi-exposure HDR fusion together with adaptive Gaussian-regularized, distance-weighted gray-centroid refinement stabilizes sub-pixel stripe localization under reflective disturbance. For challenge 2, geometry-aware contour screening and probabilistic multi-stage RANSAC fitting suppress burr-induced outliers, partial edge loss, and background interference. The stripe and contour cues are finally fused through ray–plane intersection and local depth interpolation to obtain the diameter *D* and the two datum-related center-position quantities $d_{v}$ and $d_{h}$ within the calibrated sensing volume.

The main contributions of this work can be summarized as follows:

- A coupled monocular structured-light sensing framework for metric hole inspection. By jointly organizing contour observation, structured-light depth recovery, and geometric fusion within one calibrated sensing pipeline, the proposed system provides a lightweight alternative to multi-camera three-dimensional inspection while preserving the deployment efficiency of monocular imaging.
- An HDR-assisted stripe-center-extraction strategy for reflective metallic surfaces (challenge 1). Multi-exposure radiometric fusion, adaptive Gaussian regularization, and distance-weighted gray-centroid refinement are integrated to stabilize sub-pixel stripe localization under local saturation, uneven illumination, and stripe-width variation.
- A robust circular-hole estimation module for imperfect industrial contours (challenge 2). Geometry-aware contour screening and probabilistic multi-stage RANSAC fitting are jointly used to suppress burr-induced outliers, partial edge loss, and background interference during circle-parameter estimation.
- Laboratory validation across two metallic workpieces and multiple hole diameters. Validation was conducted on a steel bolt-hole workpiece and a 6061 aluminum-alloy plate containing five holes with nominal diameters from approximately 30 to 78 mm. The experiments evaluate component-level stripe extraction, agreement with independent CMM references, and repeated dimensional measurements under a fixed calibrated configuration.

The remainder of this paper is organized as follows. Section 2 presents the measurement system, feature extraction, and structured-light-guided geometric recovery. Section 3 reports the experimental setup, validation results, and discussion. Section 4 concludes the paper.

## 2. Materials and Methods

### 2.1. Measurement System and Workflow

**Figure 1:**
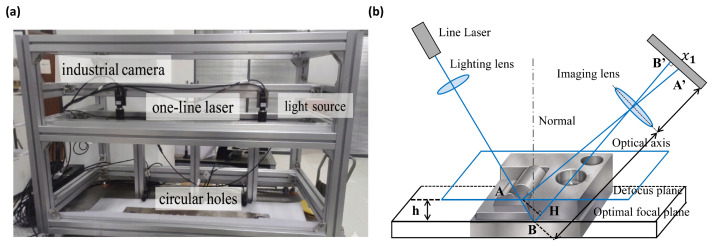
Overall sensing configuration and structured-light measurement principle for metallic circular holes. (**a**) Hardware layout of the proposed sensing platform, including the industrial camera, line laser, surface light source, and metallic workpiece. (**b**) Schematic of line-structured-light triangulation and light-plane-constrained depth recovery, where the projected stripe provides the depth cue required to compensate single-view scale ambiguity and recover the three-dimensional hole geometry.

The proposed measurement system is designed for non-contact inspection of metallic circular holes at a fixed laboratory station. As illustrated in Figure 1, the system consists of one industrial camera, one line laser, one aperture surface light, a rigid mechanical support, and a host computer for synchronized acquisition and processing. The camera is positioned above the target region to capture the global circular-hole contour, while the line laser is projected obliquely onto the metallic workpiece to provide the depth constraint. The aperture surface light enhances the visibility of the hole boundary and reduces local illumination inconsistency on reflective metal surfaces. The laboratory platform occupies an envelope of approximately 1400mm×800mm×800mm, and the typical camera-to-workpiece distance is approximately 250 mm. In this work, “compact” refers to the sensing architecture—one industrial camera, one line laser, one surface light, and a single calibrated light-plane model—rather than to a handheld or miniature instrument.

Image acquisition was performed using a HIKROBOT MV-CA050-20GC color CMOS GigE industrial camera equipped with a 16 mm, approximately F/2.4 lens. The implemented image-processing chain used 8-bit image data. A focus-adjustable MTO-Laser red line-laser module, installed using an M-H22-B mounting assembly, was operated at a center wavelength of 650 nm, a rated optical power of 50 mW, a fan angle of 60°, and a 12 V DC supply. The laser focus was adjusted for the approximately 250 mm working distance and was then fixed. The laser power or drive-current setting was kept unchanged throughout calibration and all reported experiments.

The fixed laser configuration was selected during system alignment to provide continuous line coverage over the measurement region while limiting unrecoverable saturation on the reflective workpiece. At the approximately 250 mm stand-off distance, the 60° fan angle covered the required field, and the focused stripe remained sufficiently narrow for sub-pixel center localization. Keeping the optical power, focus, camera position, and laser mounting unchanged also preserved consistency with the calibrated light-plane model.

The overall sensing principle is based on the complementary use of passive image observation and active structured-light encoding. In this architecture, the industrial camera acquires a top-view image that captures the two-dimensional boundary distribution of the circular hole, while the obliquely projected laser stripe introduces a depth-sensitive geometric cue through its spatial intersection with the target surface. The image plane is therefore used to localize the contour, whereas the structured-light cue constrains the corresponding three-dimensional geometry. This design preserves the simplicity and acquisition speed of monocular imaging while overcoming the metric ambiguity of purely two-dimensional measurement. From an implementation perspective, the workflow follows a sequential sensing-to-measurement pipeline. Raw images are first acquired under coordinated surface illumination and laser projection. The laser stripe is then localized to establish a stable geometric reference, and the circular-hole contour is extracted and refined for robust shape estimation. The fitted contour and structured-light observations are finally integrated with the calibrated imaging model and light-plane constraint to recover the actual three-dimensional parameters of the target hole, including its diameter and position-related quantities. This architectural design provides non-contact image acquisition with fewer sensing components than multi-camera configurations. The joint formulation of passive contour observation and active structured-light encoding mitigates reflective interference and removes the metric scale ambiguity of purely two-dimensional monocular measurement within the calibrated volume. The following subsections describe the stripe localization, robust feature fitting, and three-dimensional reconstruction algorithms in detail.

### 2.2. Notation and Symbol Definitions

Table 1 summarizes the main symbols used throughout the method section. Scalars are denoted by italic letters, vectors by bold lowercase letters, and matrices by bold uppercase letters. Pixel coordinates use image axes (u,v), whereas *x* denotes the one-dimensional coordinate along a stripe profile within a fixed image column. All three-dimensional quantities are expressed in the camera coordinate system unless otherwise stated.

### 2.3. Radiometric Enhancement and Robust Stripe–Hole Feature Extraction

**Figure 2:**
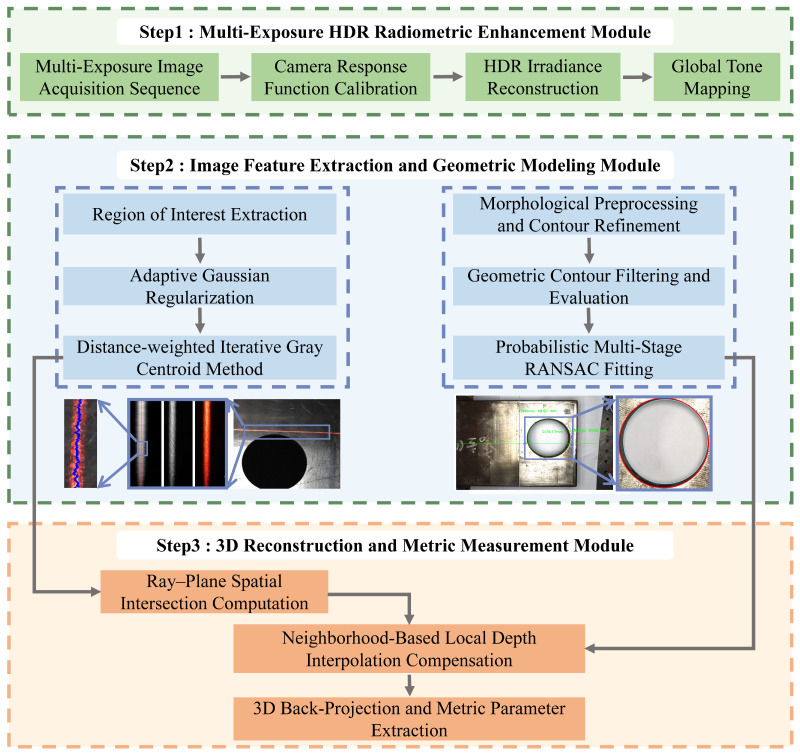
Algorithmic workflow of the proposed HDR-assisted monocular structured-light measurement framework. Step 1 performs multi-exposure HDR radiometric enhancement through exposure bracketing, camera-response calibration, HDR irradiance reconstruction, and global tone mapping. Step 2 extracts laser-stripe and circular-hole features using adaptive Gaussian regularization, distance-weighted iterative gray-centroid localization, morphological contour refinement, geometry-aware filtering, and probabilistic multi-stage RANSAC fitting. Step 3 converts the refined stripe and contour cues into 3D measurements through ray–plane intersection, local depth-interpolation compensation, back-projection, and metric parameter extraction.

The feature extraction module converts one measurement frame into two complementary outputs: a refined laser stripe centerline $\left\{u_{j}\right\}$ for depth-related geometric recovery and a robust circular-hole contour for image-domain shape estimation. As shown in Figure 2, the module contains three stages: HDR radiometric enhancement, stripe/hole feature extraction, and structured-light-guided geometric recovery. The following subsections present the symbolic formulation of each stage, and every symbol introduced in the equations is defined immediately after its first appearance.

#### 2.3.1. Multi-Exposure HDR Enhancement for Reflective Stripe Imaging

For reflective metallic surfaces, the captured laser stripe may suffer from local saturation and non-uniform intensity distribution. A single exposure cannot simultaneously preserve highlight details and weak-stripe segments. Therefore, before centerline localization, a multi-exposure HDR enhancement strategy is adopted. Let $\left\{I_{1},I_{2},\ldots ,I_{P}\right\}$ denote the captured raw image sequence, where $I_{p}$ is the image acquired under the *p*-th exposure setting, and let $\left\{t_{1},t_{2},\ldots ,t_{P}\right\}$ denote the corresponding exposure times, with $t_{1}<t_{2}<\cdots <t_{P}$. The exposure times follow a geometric progression:

(1) $$t_{j}=t_{1}r^{j-1},\;j=1,2,\ldots ,P,$$

where $t_{j}$ is the exposure time of the *j*-th frame, $t_{1}$ is the shortest exposure time, *P* is the total number of captured exposures, and r>1 is the exposure ratio controlling the geometric progression. This setting efficiently covers the wide dynamic range caused by spatially varying reflection and laser-energy attenuation.

**Figure 3:**
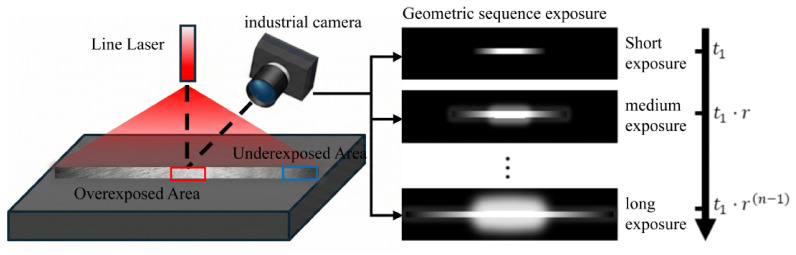
Principle of multi-exposure HDR stripe imaging on reflective metallic surfaces. A sequence of short-to-long exposures sampled in a geometric progression captures saturated and weak-response stripe regions as complementary observations, providing the radiometric basis for camera-response calibration, HDR reconstruction, and subsequent centerline extraction.

Figure 3 summarizes the acquisition principle. Short-exposure images constrain the highly reflective stripe region below saturation, whereas medium- and long-exposure images preserve stripe continuity in weak-response areas.

Because the camera response is generally nonlinear, the grayscale values from different exposure images cannot be directly averaged. Let $Z_{ij}$ denote the observed gray value at pixel *i* in exposure frame *j*, where $i=1,\ldots ,N_{\mathrm{pix}}$ indexes pixels in the stripe region and j=1,…,P. Following the classical response-calibration formulation [26], the relationship between pixel intensity, scene irradiance $E_{i}$, and exposure time is written as

(2) $$g\left(Z_{ij}\right)=\ln E_{i}+\ln t_{j},$$

where g(·) denotes the inverse camera response function, $E_{i}$ is the unknown irradiance at pixel *i*, and $t_{j}$ is the exposure time of frame *j*. The response curve and irradiance values are estimated by solving

(3) $$\begin{array}{cc} \min_{g,\{\ln E_{i}\}}\; & \sum_{i=1}^{N_{\mathrm{pix}}}\sum_{j=1}^{P}w\left(Z_{ij}\right){\left[g\left(Z_{ij}\right)-\ln E_{i}-\ln t_{j}\right]}^{2}\\ \; & +\lambda \sum_{z=Z_{\min}+1}^{Z_{\max}-1}w\left(z\right){\left[g(z-1)-2g(z)+g(z+1)\right]}^{2}, \end{array}$$

where w(·) is the intensity weighting function that down-weights near-saturated and near-black pixels, λ controls the smoothness of the response curve, and $Z_{\min}$ and $Z_{\max}$ are the minimum and maximum valid gray levels used in calibration. After calibration, the irradiance of each pixel is reconstructed from the multi-exposure sequence. Since the laser stripe is the key measurement signal, a direct averaging strategy is adopted to avoid excessive suppression of high-intensity stripe regions:

(4) $$\ln{\hat{E}}_{i}=\frac{1}{P}\sum_{j=1}^{P}\left[g\left(Z_{ij}\right)-\ln t_{j}\right].$$

where ${\hat{E}}_{i}$ is the reconstructed irradiance at pixel *i*. Global tone mapping then converts the HDR irradiance map into a low-dynamic-range image $L_{i}$ suitable for subsequent stripe-center extraction:

(5) $$L_{i}=255{\left(\frac{{\hat{E}}_{i}-E_{\min}}{E_{\max}-E_{\min}}\right)}^{1/\eta },$$

where $E_{\min}$ and $E_{\max}$ are the minimum and maximum reconstructed irradiance values in the stripe region, η is the gamma correction parameter, and $L_{i}\in \left[0,255\right]$ is the tone-mapped gray value used by the following stripe extraction module.

**Figure 4:**
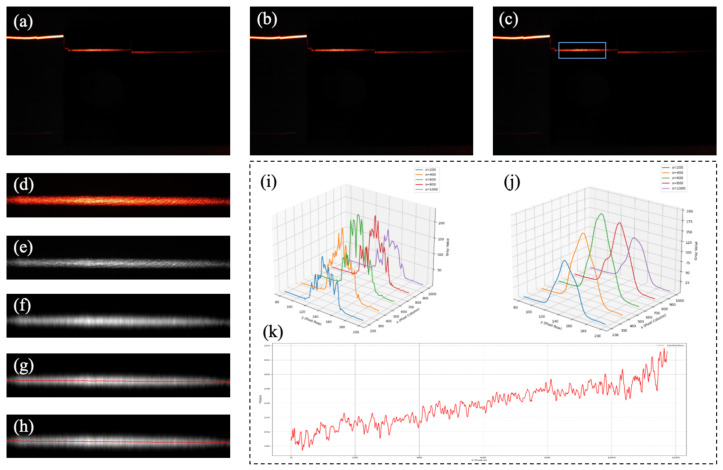
HDR-based stripe extraction and centerline refinement: (**a**) raw multi-exposure observation; (**b**) distortion-corrected image; (**c**) stripe region of interest; (**d**) raw-color laser stripe in the ROI; (**e**) HDR-fused grayscale stripe; (**f**) adaptively regularized stripe; (**g**) initial gray-centroid centerline; (**h**) final centerline after distance-weighted iterative refinement; (**i**) representative grayscale profiles before regularization; (**j**) corresponding profiles after regularization; and (**k**) final stripe-center coordinates along the image columns.

Figure 4a–f summarizes image correction, ROI localization, HDR fusion, and adaptive regularization; Figure 4g,h show the initial and refined centerlines; and Figure 4i–k show how regularization stabilizes the profile shape and the final center-coordinate sequence.

#### 2.3.2. Laser Stripe-Center Extraction

For the laser stripe branch, the tone-mapped image is restricted to a predefined region of interest (ROI) that covers the effective measurement area, so that irrelevant background responses are excluded. Let *j* index the image columns intersecting the stripe, and let $I_{j}\left(x\right)$ denote the gray-level profile along the *v*-direction within the *j*-th column, where *x* is the one-dimensional coordinate along the stripe cross-section. To reduce local noise and reflective disturbance, an adaptive Gaussian regularization is applied:

(6) $${\tilde{I}}_{j}\left(x\right)=G\left(x;\sigma_{j}\right)*I_{j}\left(x\right),$$

where ${\tilde{I}}_{j}\left(x\right)$ is the regularized profile, * denotes convolution, and $G(x;\sigma_{j})$ is a one-dimensional Gaussian kernel,

(7) $$G\left(x;\sigma_{j}\right)=\frac{1}{\sqrt{2\pi }\;\sigma_{j}}\exp\;\left(-\frac{x^{2}}{2\sigma_{j}^{2}}\right).$$

Here, $\sigma_{j}$ is the adaptive smoothing scale in column *j*, which is set proportionally to the locally estimated stripe width so that the stripe-center structure is preserved while suppressing noise.

Based on the regularized profile, a coarse stripe center is estimated to initialize the subsequent refinement. A distance-weighted gray-centroid iteration is then performed:

(8) $$u_{j}^{\left(t+1\right)}=\frac{\sum_{x}x\;w_{j}^{\left(t\right)}\left(x\right)}{\sum_{x}w_{j}^{\left(t\right)}\left(x\right)},$$

(9) $$w_{j}^{\left(t\right)}\left(x\right)={\tilde{I}}_{j}\left(x\right)\exp\;\left(-\frac{{\left(x-u_{j}^{\left(t\right)}\right)}^{2}}{2\gamma_{j}^{2}}\right),$$

where $u_{j}^{\left(t\right)}$ is the estimated stripe center in column *j* at iteration *t*, $u_{j}^{\left(t+1\right)}$ is the updated center, $w_{j}^{\left(t\right)}\left(x\right)$ is the weight assigned to profile coordinate *x*, and $\gamma_{j}$ controls the spatial attenuation around the current center estimate. The iteration stops when $|u_{j}^{\left(t+1\right)}-u_{j}^{\left(t\right)}|<\varepsilon_{u}$, where $\varepsilon_{u}$ is a preset convergence threshold. The final center set $\left\{u_{j}\right\}$ forms the sub-pixel stripe centerline used for structured-light depth recovery.

#### 2.3.3. Circular-Hole Contour Extraction and Fitting

In parallel with stripe extraction, the circular-hole branch recovers a robust contour from the same observation frame after grayscale normalization, threshold-based binarization, and morphological repair. Let ${\left\{C_{i}\right\}}_{i=1}^{N_{c}}$ denote the set of connected contour candidates. Instead of selecting the target contour by a single empirical rule, a geometry-aware score is defined for each candidate $C_{i}$:

(10) $$S\left(C_{i}\right)=\lambda_{1}\frac{|A_{i}-A_{\mathrm{ref}}|}{A_{\mathrm{ref}}}+\lambda_{2}\left(1-\frac{4\pi A_{i}}{P_{i}^{2}}\right)+\lambda_{3}\frac{d_{i}}{d_{\max}},$$

where $A_{i}$ and $P_{i}$ are the area and perimeter of candidate $C_{i}$, respectively; $A_{\mathrm{ref}}$ is the nominal contour area projected into the image domain and expressed in pixel^2^; $d_{i}$ is the distance between the candidate center and the expected measurement-region center; and $d_{\max}$ is the maximum allowable center deviation. Importantly, $A_{\mathrm{ref}}$ is not a measured diameter or a ground-truth result. It is used only as a weak product-specific prior for rejecting contours with an implausible scale. The fixed weights are $\lambda_{1}=0.15$, $\lambda_{2}=0.55$, and $\lambda_{3}=0.30$, so circularity and spatial proximity provide the dominant image evidence. The target contour is selected by

(11) $$C^{*}=\arg\min_{C_{i}}S\left(C_{i}\right).$$

After the discrete target contour has been selected, $A_{\mathrm{ref}}$ is not used in the subsequent RANSAC circle fit, structured-light reconstruction, or millimeter-scale dimensional calculation. It therefore cannot force the final measurement toward the design nominal value. Its influence on discrete contour selection is evaluated separately through the sensitivity experiment reported below.

The boundary points extracted from $C^{*}$ are denoted by ${\left\{p_{k}\right\}}_{k=1}^{N_{b}}$, where $p_{k}={\left[x_{k},y_{k}\right]}^{T}$ is the *k*-th contour point in image coordinates and $N_{b}$ is the number of retained boundary points. A probabilistic multi-stage RANSAC strategy is then used to estimate the circle center $c={\left[x_{c},y_{c}\right]}^{T}$ and radius *r*. The fitting residual of point $p_{k}$ is defined as

(12) $$\rho_{k}=\left|\;∥p_{k}{-c∥}_{2}-r\;\right|.$$

A point is considered an inlier if $\rho_{k}<\tau$, where τ is the radial residual threshold in pixels. In all experiments, τ=1 pixel, the minimum sample size is m=3, and the target confidence is p=0.99. The one-pixel threshold matches the pixel-level fluctuation of the extracted contour while rejecting larger deviations caused by burrs, specular defects, and incomplete edges. These parameters are fixed for all images and are not tuned separately for individual samples. The required number of RANSAC iterations is updated adaptively by

(13) $$N=\frac{\log(1-p)}{\log(1-w^{m})},$$

where p∈(0,1) is the target confidence level, w∈(0,1] is the current inlier ratio, and *m* is the minimum number of points required to define a circle hypothesis. The resulting iteration count is denoted by $N_{\mathrm{iter}}$. After a reliable inlier set I is obtained, the circle parameters are refined by weighted least squares:

(14) $$\min_{c,\;r}\sum_{k\in I}\eta_{k}\rho_{k}^{2},\;\eta_{k}=\exp\;\left(-\frac{\rho_{k}^{2}}{2\sigma_{r}^{2}}\right),$$

where $\eta_{k}$ is the confidence weight of the *k*-th inlier and $\sigma_{r}$ controls the influence of large-residual points during refinement. The refined center c and radius *r* provide the image-domain circular-hole model for subsequent three-dimensional recovery.

Overall, the feature extraction module outputs two stable cues for the measurement stage: a refined laser stripe centerline $\left\{u_{j}\right\}$ and a robust circular-hole contour parameterized by (c,r). Their joint use forms the input to the structured-light-guided geometric recovery described next.

### 2.4. Structured-Light-Guided Geometric Recovery

Pure monocular imaging provides two-dimensional contour information rapidly, but it cannot directly resolve the depth ambiguity required for metric three-dimensional measurement. This subsection formulates the camera model, light-plane constraint, depth assignment, and final metric extraction. All symbols are defined when they first appear.

#### 2.4.1. Camera and Light-Plane Calibration

The camera-intrinsic parameters and the laser light plane are calibrated offline using a checkerboard-based procedure. For a spatial point $P_{w}={\left[X_{w},Y_{w},Z_{w},1\right]}^{T}$ in the world coordinate system, its homogeneous image coordinate $p={\left[u,v,1\right]}^{T}$ satisfies the pinhole projection model

(15) $$s\;p=K\left[R\;t\right]P_{w},$$

where *s* is the projection scale factor, $R\in R^{3\times 3}$ is the rotation matrix from the world frame to the camera frame, $t\in R^{3}$ is the translation vector, and K is the intrinsic matrix,

(16) $$K=\left[\begin{array}{ccc} f_{x} & 0 & c_{x}\\ 0 & f_{y} & c_{y}\\ 0 & 0 & 1 \end{array}\right],$$

where $f_{x}$ and $f_{y}$ are the effective focal lengths in pixel units along the horizontal and vertical image axes, respectively, and $\left(c_{x},c_{y}\right)$ is the principal point. Lens distortion is compensated before feature extraction using the conventional radial–tangential model; the corrected pixel coordinates are then used in all subsequent geometric computations.

The line laser generates a light plane in the camera coordinate system, denoted by

(17) $$\Pi_{L}:\;aX+bY+cZ+d=0,$$

where ${\left[a,b,c\right]}^{T}$ is the unit normal vector of the light plane and *d* is the plane offset. The coefficients (a,b,c,d) are estimated offline from laser stripe observations on a planar calibration target at multiple poses.

For camera calibration, a checkerboard with a 15 mm square size and (11×8) interior corners was imaged at 20 different poses. Zhang’s calibration procedure was used to estimate the intrinsic matrix and the radial–tangential distortion parameters, and the resulting distortion correction was applied before all feature extraction. The light-plane model was then fitted from multi-pose laser observations on the calibrated target. The mean point-to-plane residual of this light-plane fit was 0.0032 mm; this value characterizes the calibration fit and is not treated as the expanded uncertainty of the complete measurement system.

#### 2.4.2. Stripe-Based Depth Recovery and Contour Back-Projection

Let $s_{j}={\left[u_{j}^{s},v_{j}^{s},1\right]}^{T}$ denote the homogeneous image coordinate of the *j*-th extracted stripe-center point. The corresponding viewing ray in the camera frame is $l_{j}\left(\mu \right)=\mu \;K^{-1}s_{j}$, where μ>0 is the depth scale along the ray. The intersection of this ray with the calibrated light plane gives

(18) $$\mu_{j}=-\frac{d}{\left[\begin{array}{ccc} a & b & c \end{array}\right]K^{-1}s_{j}},$$

(19) $$S_{j}=\mu_{j}K^{-1}s_{j}=\left[\begin{array}{c} X_{j}^{s}\\ Y_{j}^{s}\\ Z_{j}^{s} \end{array}\right].$$

where $\mu_{j}$ is the ray-depth scale of the *j*-th stripe point and $S_{j}={\left[X_{j}^{s},Y_{j}^{s},Z_{j}^{s}\right]}^{T}$ is its recovered three-dimensional coordinate in the camera frame. The depth component $Z_{j}^{s}$ serves as a local metric cue near the circular hole.

For each contour point $q_{j}={\left[u_{j},v_{j},1\right]}^{T}$, the depth is assigned by weighted interpolation over neighboring stripe samples rather than by a single pixel match. Let $N_{j}$ denote the index set of stripe points associated with contour point *j*, and let $Z_{t}=Z_{t}^{s}$ be the depth of the *t*-th neighboring stripe sample. The compensated depth is

(20) $${\hat{Z}}_{j}=\frac{\sum_{t\in N_{j}}\alpha_{jt}Z_{t}}{\sum_{t\in N_{j}}\alpha_{jt}},$$

(21) $$\alpha_{jt}=\exp\left(-\frac{{\left(u_{j}-u_{t}\right)}^{2}}{2\sigma_{z}^{2}}\right),$$

where ${\hat{Z}}_{j}$ is the interpolated depth of the *j*-th contour point, $\alpha_{jt}$ is the interpolation weight between contour column $u_{j}$ and stripe column $u_{t}$, and $\sigma_{z}$ controls the spatial decay of the weighting kernel. The three-dimensional coordinate of the contour point is then recovered by back-projection:

(22) $$Q_{j}={\hat{Z}}_{j}K^{-1}q_{j}=\left[\begin{array}{c} X_{j}\\ Y_{j}\\ Z_{j} \end{array}\right].$$

where $Q_{j}={\left[X_{j},Y_{j},Z_{j}\right]}^{T}$ is the reconstructed three-dimensional position of the *j*-th contour point.

#### 2.4.3. Metric Parameter Extraction

Let $C={\left[X_{c},Y_{c},Z_{c}\right]}^{T}$ denote the recovered three-dimensional center of the hole and let ${\left\{Q_{j}\right\}}_{j=1}^{N}$ denote the set of valid reconstructed contour points, where *N* is the number of accepted contour samples. The hole radius is estimated by

(23) $$r=\frac{1}{N}\sum_{j=1}^{N}{∥Q_{j}-C∥}_{2},$$

where *r* is the estimated radius and ${∥\cdot ∥}_{2}$ denotes the Euclidean norm. The corresponding diameter is

(24) D=2r.

**Figure 5:**
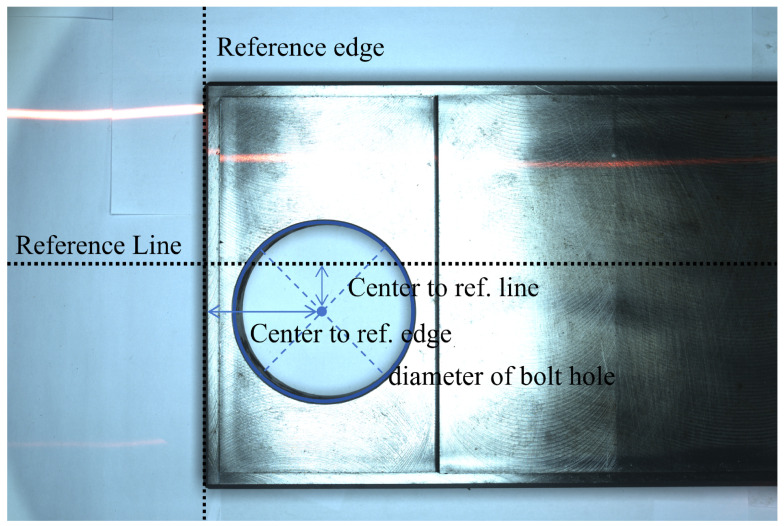
Definition of the three measured quantities and structural datums. *D* is the fitted bolt-hole diameter, $d_{v}$ is the perpendicular distance from the fitted hole center to the designated reference line $L_{v}$, and $d_{h}$ is the perpendicular distance from the fitted hole center to the designated reference edge $L_{h}$. In this figure, "ref." denotes "reference" for cross-referencing with the corresponding tables.

The three reported physical quantities are defined in Figure 5. The fitted bolt-hole diameter is D=2r. The datum $L_{v}$ is the designated straight reference line on the workpiece, and $d_{v}$ is the perpendicular distance from the fitted hole center C to $L_{v}$. The datum $L_{h}$ is the designated straight reference edge, and $d_{h}$ is the perpendicular distance from C to $L_{h}$. All three quantities are evaluated in the same reconstructed workpiece plane and datum coordinate system. The values 156, 28, and 50 mm used in the engineering drawing are design nominal values; deviations from them are reported as deviations from the design nominal, whereas absolute differences from the CMM means are used to assess agreement with an independent reference.

## 3. Results and Discussion

### 3.1. Experimental Setup and Evaluation Protocol

**Figure 6:**
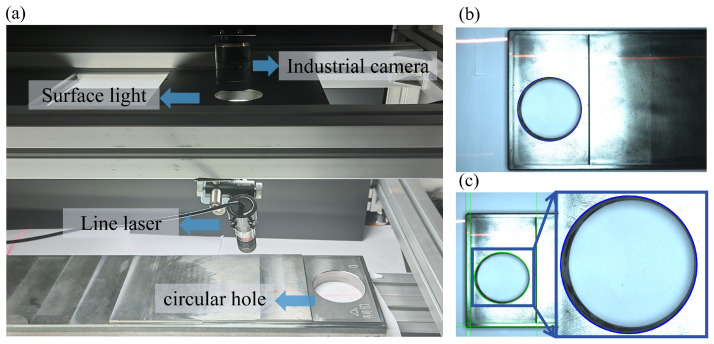
Experimental platform and representative circular-hole measurement result. (**a**) Laboratory sensing platform, showing the surface light, industrial camera, line laser, and tested circular-hole workpiece. (**b**) Captured measurement image containing the projected laser stripe and circular-hole region. (**c**) Final measurement overlay with the fitted circular contour and zoomed boundary view.

Experiments were conducted on a laboratory-built optical sensing platform for non-contact inspection of metallic circular holes. The platform integrates an industrial camera, a line laser, a surface light source, and a host computer for image acquisition and processing, with an overall envelope of approximately 1400mm×800mm×800mm and a typical camera-to-workpiece distance of approximately 250 mm. Figure 6 shows the sensing platform, a representative measurement image, and the final contour-estimation result. During data acquisition, the workpiece was positioned within the calibrated measurement region so that the hole contour and projected laser stripe were recorded in one observation frame. All repeated optical trials were acquired under a fixed workpiece placement and a fixed calibrated configuration. The reported standard deviations therefore characterize short-term repeatability under fixed fixturing rather than repeatability after workpiece removal and re-installation.

For stripe imaging under strong reflective conditions, multi-exposure acquisition was additionally used to construct the HDR-enhanced input for centerline localization. In the implemented setting, seven exposure images were captured according to the geometric progression in Equation (1), with r=2. The shortest exposure was selected to keep the high-intensity stripe region below saturation, while the longest exposure was selected to ensure that weak-stripe segments remained detectable. The reconstructed HDR image was tone-mapped into an LDR image and then processed by the proposed adaptive centerline extraction module. The auto-exposure image was retained as a visual baseline for evaluating radiometric enhancement.

The implemented acquisition and processing pipeline used 8-bit image data. For the algorithm comparison, the same seven-exposure sequence was fused once, tone-mapped to one common 8-bit grayscale HDR input, and supplied to all five stripe-center extraction methods. The methods were evaluated using the same region of interest, image columns, distortion correction, and reference construction. Thus, HDR preprocessing was not provided exclusively to the proposed center-extraction algorithm.

The values 156, 28, and 50 mm were taken from the engineering drawing and are treated as design nominal values, not as an independent ground truth. Results relative to these values are reported as deviations from the design nominal. Independent reference values were obtained using the CMM protocol described below.

The experiments were organized into three parts. First, stripe extraction was evaluated in terms of localization consistency and stability under reflective metallic-surface conditions. Second, circular-hole estimation was evaluated through the controlled synthetic robustness benchmark and the $A_{\mathrm{ref}}$ sensitivity analysis. Third, the final dimensional performance was assessed through repeated optical measurements of *D*, $d_{v}$, and $d_{h}$, reporting both deviations from the engineering-drawing design nominals and absolute differences from the independent CMM means.

For fairness and reproducibility, all images were processed under the same calibrated sensing model and fixed algorithm settings. The reported results therefore reflect the integrated performance of the proposed framework, including adaptive stripe localization, geometry-aware contour screening, robust circle fitting, and structured-light-guided geometric recovery. The following subsections first validate the key algorithmic modules and then compare the optical results with the independent CMM reference.

To extend the validation beyond one hole geometry and one material, a 6061 aluminum-alloy plate containing five circular holes was additionally measured. The holes were numbered in descending diameter order as Hole 1–Hole 5 and covered nominal diameters of approximately 78, 50, 48, 42, and 30 mm. The plate was measured using the same calibrated camera model, light-plane model, approximately 250 mm working distance, HDR acquisition sequence, and algorithm parameters used for the original steel workpiece. Three repeated optical acquisitions were processed for each hole under the fixed calibrated configuration. Only the diameter *D* was evaluated for the five-hole plate because the datum-related quantities $d_{v}$ and $d_{h}$ are defined specifically by the centerline and reference edge of the original steel workpiece.

**Figure 7:**
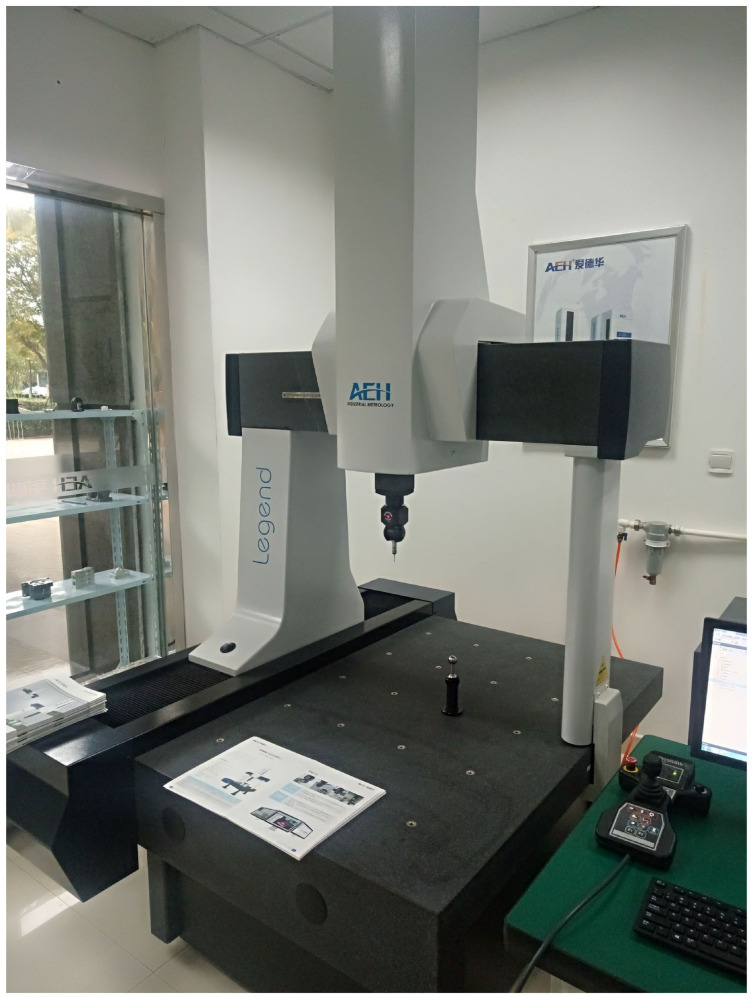
AEH Legend bridge-type coordinate-measuring machine used to obtain independent reference values for *D*, $d_{v}$, and $d_{h}$.

An AEH Legend bridge-type coordinate-measuring machine (CMM), shown in Figure 7, was used as the independent metrological reference. Its nominal maximum permissible length-measurement error was taken as ${\mathrm{MPE}}_{E}=\left(2.5+3L/1000\right)\;\mu m$, where *L* is the measured length in millimeters. For the original steel workpiece, datum plane A was fitted from nine uniformly distributed points on the main pad surface. The datum $L_{v}$ was fitted from at least five points acquired along the designated straight reference line, and the datum $L_{h}$ was fitted from at least five points acquired along the designated straight reference edge. At the same measurement height, at least 12 uniformly distributed points were acquired along the hole wall and fitted to a least-squares circle. Five repeated CMM measurements were performed to obtain independent values of *D*, $d_{v}$, and $d_{h}$ in the same datum coordinate system used for the optical measurements. For the 6061 aluminum-alloy plate, at least 12 uniformly distributed points were acquired around each hole at a common measurement height and fitted to a least-squares circle to obtain the independent diameter references.

### 3.2. Algorithm Validation

This subsection validates the two key algorithmic modules of the proposed method, namely, stripe-center extraction and circular-hole fitting. To keep the discussion focused, only component-level quantitative results are reported here. The overall runtime on practical samples and the final dimensional measurement results are presented in the next subsection.

#### 3.2.1. Effect of Multi-Exposure HDR Enhancement

**Figure 8:**
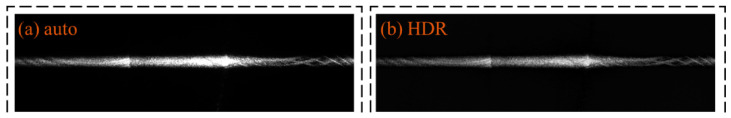
Visual comparison between auto-exposure stripe imaging and multi-exposure HDR-enhanced stripe imaging. HDR preprocessing improves the continuity of weak-stripe regions while suppressing local saturation.

Before evaluating the geometric accuracy of stripe localization, the radiometric effect of the proposed HDR preprocessing is first examined. As shown in Figure 8, the auto-exposure image contains locally saturated and intensity-imbalanced stripe regions. In contrast, the HDR-enhanced result maintains a more continuous stripe appearance while reducing the flattened bright region. This improvement is important for the following gray-centroid refinement, because a more compact and continuous stripe profile reduces the risk of center drift caused by asymmetric saturation or weak-response loss.

#### 3.2.2. Stripe-Center Extraction and Circular-Hole Fitting

Two component-level evaluations are reported below. Table 2 compares five stripe-center extraction methods using the identical tone-mapped 8-bit HDR grayscale image, region of interest, image-column range, distortion correction, and reference construction. The comparison therefore evaluates the center-extraction algorithms under a common radiometric input rather than under different acquisition conditions. Table 3 compares three circle-fitting strategies on the same controlled synthetic benchmark. The metric definitions, units, reference construction, and applicability limits are stated explicitly in the accompanying text.

For stripe validation, the proposed method was compared with the gray-centroid, geometric-center, extremum, and Steger methods. All methods received the same HDR/gray stripe input and the same region of interest. Because the ground-truth sub-pixel centerline cannot be directly observed in the real captured image, a consensus reference was constructed from the averaged result of the four baseline methods only; the proposed method was excluded from the reference construction to avoid circular evaluation. For *N* evaluated image columns, the metrics are defined as

(25) $$\mathrm{MAE}=\frac{1}{N}\sum_{j=1}^{N}\left|y_{j}-y_{j}^{\mathrm{ref}}\right|,\;\mathrm{MSE}=\frac{1}{N}\sum_{j=1}^{N}{\left(y_{j}-y_{j}^{\mathrm{ref}}\right)}^{2},\;\mathrm{RMSE}=\sqrt{\mathrm{MSE}}.$$

The reported metrics therefore quantify relative agreement with the consensus reference rather than absolute metrological truth. As summarized in Table 2, the proposed method provides a modest but consistent improvement over the gray-centroid baseline and a more pronounced improvement over the extremum and Steger methods, supporting the benefit of adaptive regularization and distance-weighted centroid refinement under non-uniform stripe intensity.

For the controlled synthetic benchmark, 100 independently generated samples were used. Each sample contained 500 points sampled from a circle with true radius $r_{\mathrm{true}}=550$ pixel, with zero-mean Gaussian perturbations of 20-pixel standard deviation and 10% uniformly distributed outliers. The center and radius errors were normalized as

(26) $$e_{c}^{\mathrm{norm}}=\frac{\sqrt{{\left(x_{c}-{\hat{x}}_{c}\right)}^{2}+{\left(y_{c}-{\hat{y}}_{c}\right)}^{2}}}{r_{\mathrm{true}}},\;e_{r}^{\mathrm{norm}}=\frac{|r_{\mathrm{true}}-\hat{r}|}{r_{\mathrm{true}}}.$$

The normalized errors are dimensionless. For the proposed method, 5.1220/550=0.0093 and 5.4804/550=0.0099. Table 3 shows that the proposed method yields the smallest center and radius errors among the compared fitting strategies. This benchmark isolates robustness to noise and outliers and does not by itself establish millimeter-scale system accuracy, which is evaluated separately using the metallic workpiece and CMM reference.

To further validate the role of $A_{\mathrm{ref}}$ as a weak screening prior, a sensitivity experiment was conducted, with the results summarized in Table 4. The nominal diameter used to generate $A_{\mathrm{ref}}$ was perturbed by −3%, −1%, 0, +1%, and +3% on five existing optical images. Because the prior is an area, $A_{\mathrm{ref}}\left(p\right)=A_{\mathrm{ref}}\left(0\right){\left(1+p\right)}^{2}$ was used, giving 25 rescoring runs while all image-derived candidate properties and weights remained fixed. All 25 runs selected the same physical hole contour, the recovered diameter remained unchanged, and the minimum best-to-second-best score margin was 0.021646. These results support the interpretation of $A_{\mathrm{ref}}$ as a weak screening prior rather than an answer-dependent constraint.

Overall, Figure 8 and the results in Table 2 and Table 3 verify that the proposed feature extraction pipeline provides radiometrically stable stripe observation, accurate stripe localization, and robust circular-hole estimation. These component-level advantages form the basis for the final system-level dimensional measurement results reported in the next subsection.

### 3.3. Measurement Performance on Metallic Circular Holes

**Figure 9:**
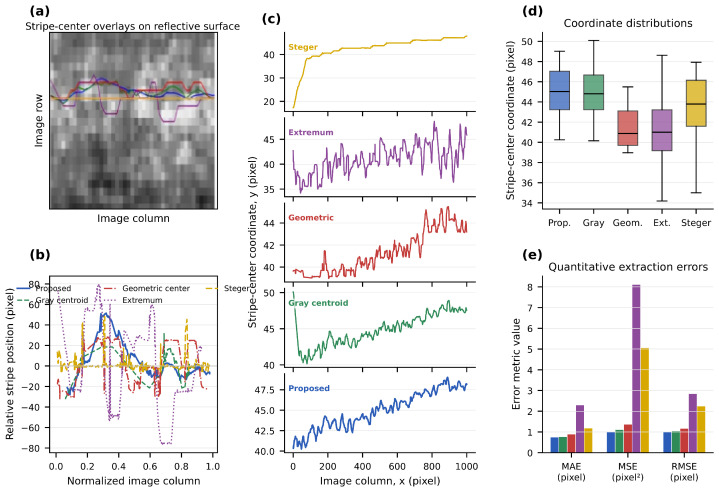
Comparison of laser stripe centerline extraction results obtained by five methods. (**a**) Extracted centerlines overlaid on the grayscale stripe image. (**b**) Centerline distributions on a white background. (**c**) Stripe-center coordinate *y* (pixel) versus image column *x* (pixel). (**d**) Boxplots of stripe-center coordinates (pixel). (**e**) Quantitative comparison using MAE (pixel), MSE (pixel^2^), and RMSE (pixel).

To quantitatively evaluate the overall measurement accuracy of the proposed method on metallic circular holes, the final dimensional results for hole diameter and position relative to structural datums are summarized in Table 5 and Table 6, along with independent CMM reference validation. To comprehensively evaluate the performance of the proposed method on metallic circular-hole inspection, we analyze both the stripe-center-extraction quality and the final dimensional measurement results. The stripe-extraction results are presented in Figure 9. In Figure 9a,b, the centerlines obtained by the five compared methods are overlaid on the grayscale stripe image and on the corresponding whiteboard background, respectively. It can be observed that the centerline produced by the proposed method follows the laser stripe more consistently and remains visually smoother than those obtained by the gray-centroid, geometric-center, extremum, and Steger methods. This indicates that the proposed extraction strategy can better suppress local fluctuation caused by image noise, surface reflection, and stripe-shape irregularity. Figure 9c further shows the coordinate variation of the extracted centerlines. Compared with the other methods, the proposed method exhibits a more stable trend and fewer abrupt deviations along the stripe direction, which is desirable for subsequent geometric reconstruction.

To make the processed numerical outputs directly available within the article, Table 7 reports the trial-level optical measurements and the corresponding fitted image-domain circle parameters for the steel workpiece.

The five repeated CMM observations used to calculate Table 6 were: D=156.034,156.036,156.035,156.037,156.033 mm; $d_{v}=28.009,28.011,28.010,28.012,28.008$ mm; and $d_{h}=50.006,50.008,50.007,50.009,50.005$ mm.

**Figure 10:**
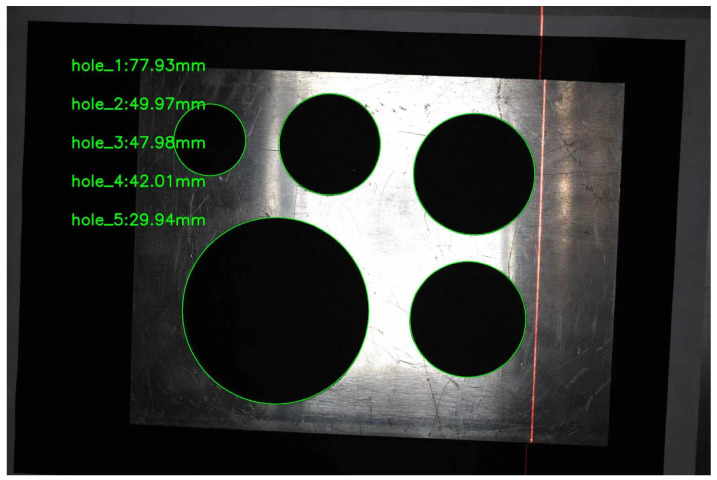
Representative multi-diameter measurement result for the 6061 aluminum-alloy test plate. The five fitted circular contours correspond to nominal diameters of approximately 78, 50, 48, 42, and 30 mm. The complete three-trial optical results and independent CMM comparisons are reported in Table 8.

As illustrated in Figure 10 for the five-hole aluminum-alloy plate, r the five-hole plate, the physical circle-center coordinates are reported in the plate coordinate system, with the upper-left plate corner defined as the origin, the positive *x*-axis directed rightward, and the positive *y*-axis directed downward. All coordinate and diameter values are expressed in millimeters.

The additional multi-diameter results are summarized in Table 8. All five holes on the 6061 aluminum-alloy plate were successfully detected and measured in the three repeated acquisitions. The mean optical–CMM absolute differences were 0.059, 0.146, 0.051, 0.006, and 0.218 mm for Hole 1–Hole 5, respectively. The corresponding standard deviations were 0.080, 0.550, 0.293, 0.230, and 0.176 mm. Across the five holes, the average absolute difference was 0.096 mm and the RMSE was 0.122 mm. The variation among holes shows that measurement performance is influenced by contour scale, local reflection, and image coverage; therefore, the high agreement obtained for the original steel hole should not be extrapolated uniformly to every diameter and material.

The statistical distribution of the extracted centerlines is illustrated in Figure 9d. The proposed method produces a compact distribution with fewer large local deviations. Because the true sub-pixel stripe center cannot be directly observed, the mean of the four baseline methods is used only as a consensus reference, and the MAE, MSE, and RMSE in Figure 9e quantify relative consistency rather than absolute metrological accuracy. The proposed method yields the lowest values for all three consistency metrics, indicating a stable geometric cue for the subsequent dimensional recovery stage under the tested reflective-surface condition.

Table 5 compares the passive monocular, standard active, and proposed pipelines using the engineering-drawing values as design nominals. All three schemes share the same hardware, calibration model, and measurement protocol and differ only in the algorithmic pipeline. Passive monocular (2D) uses image-domain circle fitting without structured-light depth recovery. Standard active (Laser + LS) uses conventional gray-centroid stripe extraction and direct least-squares circle fitting, whereas the proposed method uses the complete HDR-assisted, geometry-aware, probabilistic RANSAC, and structured-light reconstruction pipeline. For *D*, the proposed method gives 156.04±0.03 mm and a maximum deviation of 0.09 mm from the 156 mm design nominal, compared with 1.82 mm and 0.72 mm for the passive and standard-active baselines. These design-nominal deviations describe conformity to the drawing and are not used as independent accuracy evidence.

For $d_{v}$, the proposed method gives 28.00±0.01 mm, with a maximum deviation of 0.02 mm from the 28 mm design nominal; for $d_{h}$, it gives 50.01±0.02 mm, with a maximum nominal deviation of 0.04 mm. The corresponding passive/standard-active maximum nominal deviations are 0.42/0.18 mm for $d_{v}$ and 0.75/0.30 mm for $d_{h}$. Independent agreement is evaluated in Table 6: the absolute differences between the proposed optical means and the CMM means are 0.005 mm for *D*, 0.010 mm for $d_{v}$, and 0.003 mm for $d_{h}$. Within the tested fixed configuration, these comparisons support the repeatability of the proposed pipeline and its agreement with the independent reference.

### 3.4. Measurement Uncertainty Considerations

The main uncertainty sources are camera-intrinsic and distortion calibration, camera-to-workpiece alignment, light-plane calibration, stripe-center localization, contour segmentation, robust circle fitting, local depth interpolation, and fitting of the designated reference line and reference edge. Camera calibration errors perturb the pixel-ray direction and therefore affect all reconstructed coordinates. Light-plane calibration errors perturb the ray–plane intersection; the reported 0.0032 mm value describes the mean point-to-plane residual of the light-plane fit and is not the expanded uncertainty of the complete measurement system. Stripe-center localization and contour segmentation errors are amplified by specular saturation, edge defects, and incomplete contours. The fitted diameter *D* is mainly affected by the reconstructed hole contour and circle-fitting residuals, whereas $d_{v}$ and $d_{h}$ additionally include uncertainty from the corresponding datum-line fits.

(27) $$u_{c}\left(y\right)=\sqrt{\sum_{i}{\left(\frac{\partial y}{\partial x_{i}}u\left(x_{i}\right)\right)}^{2}},\;y\in \left\{D,d_{v},d_{h}\right\}.$$

The standard deviations reported for the repeated optical and CMM measurements describe repeatability under their stated fixed setups and should not be interpreted as a complete expanded uncertainty budget. The CMM comparison supplies an independent reference, while the uncertainty discussion identifies the principal error-propagation paths of the optical measurement chain.

### 3.5. Discussion

Under the tested laboratory configuration, the proposed method provides a favorable balance between repeatability, robustness, and acquisition efficiency. Multi-exposure HDR preprocessing preserves weak-stripe segments and suppresses local saturation; line-structured light supplies the depth constraint needed for metric reconstruction within the calibrated measurement volume; and adaptive stripe-center extraction and robust circular-hole fitting reduce sensitivity to local defects and background interference. The CMM comparison provides an independent reference for the final three quantities, while Table 9 clarifies the different application conditions. The optical system supports rapid non-contact acquisition at a fixed station but depends on radiometric control and calibration stability; the CMM provides stronger metrological traceability but requires a controlled environment and sequential probing.

Comparison with prior studies should be interpreted in the context of different metrological tasks. Circle-structured-light inner-surface systems have reported a diameter absolute error below 3 μm, a mean relative error of 0.015%, or a relative error below 0.012% under their respective calibration and scanning configurations [18,19,20]. Binocular circular-hole systems have reported errors or standard deviations at the level of several tens of micrometers [6,7], while a line-structured-light method for reflective metallic surfaces reported a 0.025 mm average height error and 0.026 mm repeatability on a standard block [14]. In the present work, the original steel workpiece showed close agreement with the CMM for the three designated quantities, whereas the added 6061 aluminum-alloy plate produced mean-diameter differences of 0.006–0.218 mm over five holes. Because the compared studies use different specimens, measurands, sensing geometries, working distances, calibration procedures, and uncertainty definitions, these values should not be treated as a direct performance ranking. The contribution of the present system is the integration of HDR-assisted reflective-stripe observation, calibrated light-plane reconstruction, robust external hole-contour fitting, and datum-related dimensional extraction using one camera and one line laser.

Multi-exposure HDR and single-frame high-bit-depth acquisition address radiometric range in different ways and should not be regarded as strictly equivalent. A 12- or 14-bit camera records finer-intensity quantization within one exposure and can reduce acquisition latency and motion sensitivity. Multi-exposure HDR combines frames acquired at different exposure times to preserve both weak and strongly reflected stripe regions, but it increases acquisition time and assumes limited scene motion. The retained dataset and implemented processing chain in this study contain 8-bit images, and no matched 12/14-bit raw-image experiment was acquired. Consequently, the present results demonstrate the benefit of HDR preprocessing only within the tested multi-exposure configuration and do not establish that it is superior to high-bit-depth single-frame imaging.

The reported results were obtained using one fixed 650 nm, 50 mW line-laser configuration with a 60° fan angle and an approximately 250 mm working distance. Laser power, fan angle, projection geometry, focus, and stand-off distance were not independently varied. The present experiments therefore cannot isolate the sensitivity of the final dimensional error to any single laser parameter. A controlled multi-parameter study would be required before transferring the reported performance to substantially different laser modules or installation geometries.

The revised validation includes one steel bolt-hole workpiece and one 6061 aluminum-alloy plate containing five additional hole diameters. This extension demonstrates successful measurement over the tested nominal diameter range of approximately 30–156 mm and two metallic materials, but it does not establish unrestricted generalization. Material reflectance, roughness, coatings, oxidation, contamination, hole chamfer, and contour scale can still change stripe saturation, edge completeness, and depth interpolation. All repeated optical measurements were acquired without removing and re-installing the workpiece; re-fixturing repeatability and the associated datum-reconstruction error were therefore not evaluated. The geometric recovery also depends on offline calibration, installation stability, and the local coplanarity assumption. In addition, because $A_{\mathrm{ref}}$ is a product-specific weak prior, a true hole whose size lies far outside the expected product range could be incorrectly penalized. The current contour-screening strategy is therefore intended for inspection of known product families rather than unconstrained search for completely unknown hole sizes. Additional materials, surface treatments, non-circular defects, and repeated workpiece re-fixturing remain outside the scope of the present study.

## 4. Conclusions

This paper presented a non-contact measurement method for metallic circular holes by combining one industrial camera with line-structured light. Multi-exposure HDR enhancement under the fixed static acquisition condition, adaptive stripe-center extraction, geometry-aware contour screening, and probabilistic multi-stage RANSAC fitting were integrated with calibrated light-plane reconstruction. Validation on a steel bolt-hole workpiece and a 6061 aluminum-alloy five-hole plate covered tested nominal diameters of approximately 30–156 mm. The original steel workpiece agreed with the independent CMM reference values within 0.005 mm for *D*, 0.010 mm for $d_{v}$, and 0.003 mm for $d_{h}$, while the five added hole diameters produced mean optical–CMM diameter differences of 0.006–0.218 mm. These results support the feasibility of the integrated sensing pipeline over the tested materials, hole sizes, and fixed calibrated configuration. They do not establish superiority over 12/14-bit single-frame acquisition or generalization to other laser configurations. Broader claims require validation across additional materials, surface finishes, hole geometries, stand-off distances, laser parameters, and workpiece re-fixturing conditions.

## Figures and Tables

**Table 1:** Main notation used in the proposed measurement method.

Symbol	Description
$I_{j}$, $I_{j}\left(x\right)$	HDR-enhanced image and gray profile in the *j*-th image column
$I_{p}$, $t_{j}$, *P*, *r*	Raw exposure image, exposure time, number of exposures, exposure ratio
$Z_{ij}$, $E_{i}$, ${\hat{E}}_{i}$, $L_{i}$	Observed gray value, irradiance, reconstructed irradiance, tone-mapped LDR image
$N_{\mathrm{pix}}$, $Z_{\min}$, $Z_{\max}$	Number of stripe pixels, min/max valid gray levels in calibration
g(·), w(·), λ, η	Inverse camera response, intensity weight, smoothness weight, gamma parameter
$G(x;\sigma_{j})$, $\sigma_{j}$, ${\tilde{I}}_{j}\left(x\right)$	Gaussian kernel, adaptive scale, regularized stripe profile
$u_{j}$, $u_{j}^{\left(t\right)}$, $w_{j}^{\left(t\right)}\left(x\right)$, $\gamma_{j}$, $\varepsilon_{u}$	Stripe center, center at iteration *t*, pixel weight, spatial decay, convergence threshold
$N_{c}$, $C_{i}$, $A_{i}$, $P_{i}$, $d_{i}$, $S(C_{i})$	Number of contour candidates, candidate, area, perimeter, center distance, score
$A_{\mathrm{ref}}$, $d_{\max}$, $\lambda_{k}$	Projected nominal contour area in pixel^2^, max center distance, screening weights
$N_{b}$, $p_{k}$, c, *r*, $\rho_{k}$	Number of boundary points, contour point, circle center, radius, residual
τ, *p*, *w*, *m*, $N_{\mathrm{iter}}$, I, $\eta_{k}$, $\sigma_{r}$	Inlier threshold, confidence, inlier ratio, sample size, RANSAC iterations, inlier set, weight, refine scale
*s*, R, t, $f_{x}$, $f_{y}$, $c_{x}$, $c_{y}$	Projection scale, rotation, translation, focal lengths, principal point
K, $s_{j}$, $q_{j}$, $S_{j}$, $Q_{j}$	Intrinsic matrix, stripe image point, contour image point, 3D stripe point, 3D contour point
$\Pi_{L}$, a,b,c,d, $\mu_{j}$, ${\hat{Z}}_{j}$	Light plane, plane coefficients, ray-depth scale, interpolated depth
$N_{j}$, $\alpha_{jt}$, $\sigma_{z}$	Neighboring stripe set, interpolation weight, depth-decay scale
C, *N*, *D*, $X_{j},Y_{j},Z_{j}$	Recovered 3D hole center, number of 3D contour points, diameter, contour coordinates
$L_{v}$, $L_{h}$, $d_{v}$, $d_{h}$	Reference line, reference edge, and center-to-datum distances

**Table 2:** Comparison of laser stripe-center extraction methods. MAE and RMSE are reported in pixel, whereas MSE is reported in pixel^2^.

Method	MAE (pixel)	MSE (pixel^2^)	RMSE (pixel)
Gray centroid	0.773	1.108	1.053
Geometric center	0.894	1.365	1.168
Extremum	2.299	8.115	2.849
Steger	1.184	5.067	2.251
Proposed	0.749	0.995	0.998

**Table 3:** Comparison of circle-fitting methods on the controlled synthetic benchmark. Center and radius errors are reported in pixel; normalized errors are dimensionless.

Method	Center Error (pixel)	Normalized Center Error	Radius Error (pixel)	Normalized Radius Error
Least squares	19.9661	0.0363	42.7919	0.0778
PSO fitting	88.7233	0.1613	105.4225	0.1916
Proposed	5.1220	0.0093	5.4804	0.0099

**Table 4:** Sensitivity of contour selection to perturbations of the nominal diameter used to generate $A_{\mathrm{ref}}$.

Diameter Perturbation	Consistent Selections	Max. ΔD (mm)	Min. Score Margin
−3%	5/5	0.000000	0.043410
−1%	5/5	0.000000	0.041778
0%	5/5	0.000000	0.040999
+1%	5/5	0.000000	0.034406
+3%	5/5	0.000000	0.021646

**Table 5:** Bolt-hole dimensional measurements relative to the engineering-drawing design nominal values under passive monocular, standard active, and proposed methods.

Measured Quantity	Trial No.	Design Nominal (mm)	Passive Monocular (2D)		Standard Active (Laser + LS)		Proposed Method	
			Meas. (mm)	Rel. Err (%)	Meas. (mm)	Rel. Err (%)	Meas. (mm)	Rel. Err (%)
Hole diameter, *D*	1	156	157.42	0.91	156.72	0.46	156.02	0.01
	2		157.38	0.88	156.65	0.42	156.05	0.03
	3		157.82	1.17	156.68	0.44	156.01	0.01
	4		157.01	0.65	156.71	0.46	156.09	0.06
	5		156.82	0.53	156.70	0.45	156.03	0.02
	Mean ± s.d.		157.29 ± 0.39	—	156.69 ± 0.03	—	156.04 ± 0.03	—
	Max. nominal deviation		1.82	—	0.72	—	0.09	—
Center-to-reference line, $d_{v}$	Mean ± s.d.	28	28.35 ± 0.04	—	28.12 ± 0.03	—	28.00 ± 0.01	—
	Max. nominal deviation		0.42	—	0.18	—	0.02	—
Center-to-reference edge, $d_{h}$	Mean ± s.d.	50	50.62 ± 0.05	—	50.22 ± 0.03	—	50.01 ± 0.02	—
	Max. nominal deviation		0.75	—	0.30	—	0.04	—

**Table 6:** Independent CMM reference measurements and comparison with the proposed optical method. Values are mean ± standard deviation over five repeated measurements.

Quantity	Design Nominal (mm)	CMM (mm)	Proposed Optical (mm)	|Δ| from CMM (mm)	CMM Repeatability (mm)
Hole diameter, *D*	156.000	156.035 ± 0.002	156.040 ± 0.030	0.005	0.002
Center-to-reference-line, $d_{v}$	28.000	28.010 ± 0.002	28.000 ± 0.010	0.010	0.002
Center-to-reference-edge, $d_{h}$	50.000	50.007 ± 0.002	50.010 ± 0.020	0.003	0.002

**Table 7:** Trial-level optical measurements and fitted image-domain circle parameters for the steel bolt-hole workpiece.

Trial	$x_{c}$ (pixel)	$y_{c}$ (pixel)	*r* (pixel)	*D* (mm)	$d_{v}$ (mm)	$d_{h}$ (mm)
1	732.2793	371.3697	165.6024	156.02	28.016	50.009
2	802.0000	406.4521	161.8344	156.05	27.989	49.986
3	798.9312	406.8142	169.3995	156.01	28.000	50.015
4	572.4188	519.5498	157.7864	156.09	27.997	50.040
5	806.6927	428.3869	157.5710	156.03	27.998	50.000

**Table 8:** Complete three-trial optical measurements, plate-coordinate circle centers, and independent CMM references for the 6061 aluminum-alloy five-hole plate.

(**a**) Plate coordinates and three repeated optical measurements					
**Hole ID**	**x (mm)**	**y (mm)**	**Trial 1 (mm)**	**Trial 2 (mm)**	**Trial 3 (mm)**
Hole 1	60	100	77.84	77.93	78.00
Hole 2	140	40	49.73	49.97	50.78
Hole 3	140	100	47.81	47.98	48.38
Hole 4	80	30	41.79	42.01	42.25
Hole 5	30	30	29.59	29.94	29.79
(**b**) Statistical summaries and independent CMM comparisons					
**Hole ID**	**Optical Mean (mm)**	**SD (mm)**	**CMM Reference (mm)**	**Absolute Difference (mm)**	
Hole 1	77.923	0.080	77.982	0.059	
Hole 2	50.160	0.550	50.014	0.146	
Hole 3	48.057	0.293	48.006	0.051	
Hole 4	42.017	0.230	42.011	0.006	
Hole 5	29.773	0.176	29.991	0.218	

**Table 9:** Deployment-condition comparison between the proposed optical system and the CMM reference.

Criterion	Proposed Optical System	AEH Legend CMM
Measurement mode	Non-contact, single-camera structured light	Contact probing with traceable coordinate measurement
Hardware/space	One camera, one line laser, one surface light; 1400×800×800 mm platform envelope	Floor-standing bridge-type metrology system
Typical workflow	Parallel image acquisition and batch geometric reconstruction	Sequential mechanical probing of datum and hole features
Strength	Rapid fixed-station inspection without probe contact	Independent reference and stronger metrological traceability
Main limitation	Accuracy depends on camera/light-plane calibration and reflection suppression	Requires a controlled metrology environment and longer point-by-point measurement

## Data Availability

The processed numerical results supporting the findings of this study are provided within this article. These data include the trial-level optical and CMM measurements, fitted image-domain circle centers and radii, physical center coordinates and repeated diameter measurements for the five-hole plate, stripe-center comparison metrics, $A_{\mathrm{ref}}$ sensitivity results, and circle-fitting comparison results. The raw experimental images are not publicly available because of intellectual-property restrictions.
